# Reduced Occurrence of Gastrointestinal Symptoms in Chinese Infants Fed Minimally Processed Commercially Available Formula: A Cross-Sectional Observational Study

**DOI:** 10.1155/2020/1807397

**Published:** 2020-03-25

**Authors:** Xiao Yang Sheng, Vanitha Buthmanaban, Glenn A. A. van Lieshout, Panam Parikh

**Affiliations:** ^1^Xinhua Hospital, Shang Jiaotong University School of Medicine, Shanghai, China; ^2^Shanghai Institute for Pediatric Research, School of Medicine, Shanghai Jiao Tong University, Shanghai, China; ^3^FrieslandCampina, Amersfoort, Netherlands

## Abstract

Healthy Chinese infants consuming one of four commercially available infant formulas (IF) were assessed on the occurrence of gastrointestinal symptoms associated with suboptimal digestion of processed milk proteins. The IF differed in blocked lysine (BL) levels, a proxy indicator of heat processing as well as the nutritional quality of milk. A cross-sectional, observational study of one week was conducted in healthy, term, exclusively formula-fed Chinese infants (*n* = 452) fed with one of four commercially available IF (IF A *n* = 106, BL 9%; IF B *n* = 119, BL 12%; IF C *n* = 113, BL 11%; IF *D n* = 114 BL 20%). Parents/caretakers were requested to report feeding quantity, gastrointestinal symptoms, crying behavior, and stool characteristics daily using subject dairy and Amsterdam Infant Stool Scale (AISS). Infants fed with IF A reported less “hard” and “watery” stools and more “soft/formed” stools. Higher percentages of score I (yellow/golden) or II (orange) and less green (score III) coloured stools were noted for IF A-fed infants compared to all other formulas according to AISS. Night time crying was also significantly lower in the IF A groups compared to all other formulas. Furthermore, a higher percentage of parents/caretakers of IF A-fed infants reported absence or no complaints of abdominal distension, burping, flatulence, diarrhea, and constipation. Results suggest lower occurrence of GI symptoms and lower crying time at night in infants fed with minimally processed formula (indexed by BL levels). Future studies are required to confirm the association between minimal processing of milk formula and improved gut comfort in healthy infants.

## 1. Introduction

During the first months of life, the gastrointestinal (GI) system of infants strives to adapt itself to various nutrients in order to perfect its digestive, absorptive, and immunological functions [1]. During this “stressful” period, infants may frequently suffer from transient GI disorders and this may cause considerable discomfort to the infant and to the caregivers [2]. In a prospective, population-based study, Iacono et al. observed that 55% of healthy infants younger than six months suffer from at least one GI symptom [1]. Regurgitation, colic, and constipation have been reported as some of the common GI complaints in this age group [1, 3, 4].

Although the aetiology of GI symptoms is not fully understood, there are indications that it can relate to gestational age, birth weight, the type of milk the infant receives, microbiota composition, and even parent perception [2, 5]. Of the various GI problems, “constipation” and/or “hard stools” are often associated with formula feeding [1, 6]. In contrast, breastfed term infants are consistently reported to have softer stools for at least the first 20 weeks of life [6]. These differences between breast- and formula feeding have been recognized since early days and still remain, despite improvements in the nutrient composition and manufacture of infant formulas [6]. This suggests that besides the composition of infant formula, the way it has been processed is also a crucial determinant for its quality and in the management of GI discomfort in healthy infants.

For the production of high-quality infant formula, ensuring minimal damage to the sensitive ingredients while maintaining microbiological safety is crucial [7]. Infant formula belongs to the class of products which requires optimal quality and availability of nutrients, because it is often the only nutrient source for infants. Consequently, it must be guaranteed that infant formula contains, in sufficient quantity, all nutrients that are necessary for healthy growth and development. The availability of the essential amino acid lysine is one of the key indicators determining the nutritive value of infant formula. Lysine is biologically available only if its *ε*-amino group is free [7–10]. Heat processing of infant formula triggers a reaction between the *ε*-amino group of lysine and lactose (Maillard reaction) which renders lysine unavailable for protein synthesis and reduces the amount of bioavailable or “reactive” lysine [7, 11, 12]. This reaction mostly occurs during spray-drying and storage of milk powder or infant formula and the loss of lysine availability is mainly related to the spray-drying temperatures used [7, 13]. Finot et al. were the first to prove that the Amadori product of lysine, namely, Ne-fructosyllysine, was not used as a lysine source *in vivo* [14]. From that time on, the quantification of ‘‘lysine blockage” or “blocked lysine (BL)” due to the early Maillard reaction has been a widely used tool to assess the nutritional quality of processed milk products [11, 12, 15, 16]. Furthermore, acknowledging these untoward effects of heat treatment during infant formula processing on the nutritional value of protein, the European Food Safety Authority (EFSA) has recommended “the contents of Maillard reaction products and protein degradation products be kept as low as technologically possible” [15].

Maillard reaction products are also known to prevent proteases such as trypsin and carboxypeptidase from acting on the protein by blocking enzymatic cleavage sites, thereby decreasing the digestibility of the protein [12, 17]. In infants, the structure and function of the gut, as well as digestive enzyme production in the gut and pancreas still needs to be fully developed [18–20]. Incompletely digested nutrients might, therefore, enter the colon and be fermented by the (also developing) colonic microbiota, yielding excessive gas formation [2] and potentially causing GI discomfort [21]. In some Asian countries like China where Traditional Chinese Medicine (TCM) is still widely practiced, this gas formation, including the presence of GI discomfort (e.g., constipation, abdominal distension) is often explained as imbalance of “qi” or “chi” and is accepted as a physical manifestation of “heatiness” or “yang” (Pacific College, Accessed 22/03/2019).

Although these GI problems are usually considered minor and not harmful for the baby [2], given the distress when present for both the affected infants and their caregivers, prevention or management of GI symptoms through a nutritional approach is considered relevant and desirable [2, 22]. In view of the above, in an exploratory study, we assessed the stool characteristics, the occurrence of GI symptoms and crying patterns of Chinese infants exclusively consuming one of four commercially available infant formulas.

## 2. Methods

### 2.1. Participants and Study Area

Healthy, term infants (gestational age 37–42 weeks and birth weight 2.5–4.0 kg), aged 0–4 months, exclusively fed with a commercially available formula for at least 1 week prior to the study were recruited from paediatric clinics in Shanghai, Shenyang, and Beijing (screened *n* = 1326; enrolled *n* = 452). Infants were assigned to one of the four arms based on the formula currently in use: IF A (FrieslandCampina), IF B, IF C, and IF D. A total of 409 infants completed the study (IF *A* = 106, IF *B* = 119, IF *C* = 113, IF *D* = 114). All infants included in the study were considered for baseline analysis and those who completed the study were considered for endpoint comparison. Of the 43 infants who did not complete the study, 31 were lost to follow-up (IF *A n* = 1, IF *B n* = 14, IF *C n* = 8, IF *D n* = 8) and 12 were withdrawn due to noncompliance (IF *B n* = 5, IF *C n* = 2, IF *D n* = 5). Infants were included in the study if there was written informed consent from the parents/legal guardians. Infants were excluded if they were on breastfeeding (partial or full) or received complementary feeding, with any congenital abnormalities, using antibiotics or TCM and/or with a known or higher risk of milk allergy or lactose intolerance. Infants were also excluded if they were participating in any other nutrition intervention.

### 2.2. Study Design

The study was designed as a seven-day, cross-sectional, observational study with exclusively formula-fed infants allocated to one of four groups based on the existing use of infant formula. The infant formulas included in the study were selected based on their BL levels, a well-accepted proxy indicator for heat processing [23]. Total lysine content and furosine content after acid hydrolysis was measured using ion-pair reversed-phase HPLC (Ansynth Service B.V., Breda, the Netherlands) (Delgado et al., 1992). The furosine content was then used to calculate the level of lysine blockage in the samples [12]. The composition of the consumed infant formulas as available on the labels and the analyzed BL levels are listed in Table 1. None of the formulas included in this study contained probiotics. The primary objective was to assess the occurrence of gastrointestinal symptoms, crying behavior, and stool characteristics in infants consuming these different infant formulas. This study was approved by the Institutional Ethics Review Board of the Shanghai Nutrition Society and was performed in accordance with the ethical standards laid down in the 1964 Declaration of Helsinki and its later amendments (October 1996 amendment). The study is registered in the Netherlands Trial Registry NL5970/NTR6344.

### 2.3. Formula Consumption

The parents and/or caregivers were encouraged/requested to continue formula feeding per practice and follow the feeding table as per label instructions. Time, amount, and volume of formula consumed by the infants per day were recorded for all seven days in a subject diary by parents and/or caregivers.

### 2.4. Stool Characteristics, Gastrointestinal Symptoms, and Crying Patterns

For all seven days, parents and/or caregivers were asked to record gastrointestinal symptoms in the subject diary and stool characteristics assessment of the first defecation was performed using the validated Amsterdam Infant Stool Scale (AISS) [25]. AISS assesses the consistency (four categories: watery, soft, formed, and hard), amount/volume (smear to more than 50% of the nappy's surface), and colour (six categories from light to dark) of stools. An average score of 7-day parental reports for consistency, amount/volume, and colour was calculated. Parents and/or caregivers also maintained a crying log. Duration of crying, the pattern of crying/fussy behavior (sleeping; awake, satisfied; awake, fussy; and awake, crying), and time of the day (morning, afternoon, evening, and night where morning = 6 am-noon, afternoon = noon-6 pm, evening = 6 pm-midnight, and night = midnight-6 am) were recorded in the subject diary. The mean crying duration per day and the pattern of crying during the day were recorded.

### 2.5. Heatiness

“Clinical manifestations of infants and young children heatiness” questionnaire was developed in collaboration with a TCM practitioner and piloted in this study. This questionnaire assessed heatiness (four categories: 0–20 no heatiness; 20–50 mild heatiness; 50–80 moderate heatiness; 80–100 severe heatiness) based on individual symptoms such as dry faeces, sleeping problems, eye boogers, palm temperature, dry cough, bleeding nose, dry mouth. Parents/caregivers were asked to assign a score for three categories (none/normal, sometimes, and often) of individual heatiness symptoms. The total score of heatiness was calculated as the sum of the individual scores for all heatiness symptoms.

### 2.6. Statistics

A minimum sample size of 100 infants for each group was considered to be sufficient to see differences in gastrointestinal symptoms and stool characteristics between groups. Assuming a dropout rate of 10%, it was aimed to enroll about 440 infants in the study. Descriptive statistics (mean + SD, ranges, and percentages) are presented for baseline measurements. Between-group comparisons at baseline for normally distributed data were done using ANOVA. For data that were not normally distributed, a Kruskal–Wallis test was done. All infants included into the study and having completed two measurements (baseline and end of study) were included for analysis of time point comparison. The effect of the product consumed was tested using type 3 General Estimation Equation (GEE) analysis applying multinomial logistic regression for repeated measurements and pairwise odds ratio (OR) for significant outcomes. The crying parameters were tested using repeated measure ANOVA. All models for repeated measures were adjusted for covariates: study site, amount of product consumed, gender, caesarean section, and age at inclusion. Bonferroni adjustment was applied for multiple comparisons among study products. Correlation analyses were performed by Spearman's rho test taking along all participants. All statistical analyses were performed using SAS version 9.3 (SAS Institute Inc., Cary, USA). Statistical significance was inferred at *p* < 0.05.

## 3. Results

### 3.1. Baseline Characteristics

The four groups (IF *A*–IF *D*) were comparable for age, gender, anthropometric indices, Apgar score, parental education, and socioeconomic status (Table 2). Formula feeding was initiated at approximately 8–10 weeks after birth. The feeding frequency and amount were about 6 times per day and 725 ± 93 ml per day for all groups (Table 3). No significant differences were observed amongst the four groups for formula feeding start age, daily feeding frequency, daily feeding amount, and main caregiver.

### 3.2. Stool Consistency and Stool Colour

No significant differences were reported in daily stool frequency and stool amount between groups (Table 4). In contrast, the stool consistency of the first defecation of each day differed based on the product consumed. There were higher reports of soft/formed stools and less for watery or hard stools from the IF *A* group compared to infants fed IF *B*, IF *C* or IF *D*. The stool colour of the first defecation of each day of infants fed IF *A* also differed compared to IF *B* (*p* < 0.0001), IF *C* (*p*=0.0006), and IF *D* (*p*=0.008) (Tables 4 and 5). A higher percentage of IF *A*-fed infants reported score I (yellow/golden) or II (orange) for stool colour and less green stool (score III) compared to other IF fed groups (Table 4). Differences in stool colour were also reported for IF *B* vs IF *C* (*p*=0.002) and IF *D* (*p*=0.0005) (Table 5) whereby IF *B* had lower reports of score II (orange) stools.

### 3.3. Gastrointestinal Symptoms

The occurrence of gastrointestinal symptoms per product group is presented in Table 4. For all seven days, absence or no complaints of abdominal distension, burping, flatulence, diarrhea, and constipation were reported by a higher percentage of parents and/or caregivers for IF *A*-fed infants compared to other IF fed groups. Further analysis showed that infants fed IF *A* had lower odds for abdominal distension, burping, flatulence, diarrhea, and constipation compared to IF *B*, IF *C,* and IF *D* (Table 5). Additionally, IF *D* fed infants had a higher odds of flatulence than IF *C* (*p*=0.0002 and *p*=0.033) while IF C-fed infants were reported to be more prone to constipation (*p*=0.0001 and *p*=0.033) than those consuming IF B and IF *D* (Tables 4 and 5). There was no difference in the occurrence of colic, regurgitation, and vomiting based on the consumed product (Table 4).

A Spearman's rho test was performed to check for the correlation between all parameters measured in the diaries. This showed a correlation between stool consistency score and stool amount (*ρ* = 0.26; *p*=0.001), stool colour (*ρ* = 0.16; *p*=0.001), and a mild negative correlation with GI symptoms of abdominal distension, burping, flatulence, and diarrhea (*ρ* all∼0.10 with *p*=0.001). Several GI symptoms (abdominal distension, burping, flatulence, diarrhea, colic, diaper dermatitis, and back arching) correlate with each other (*ρ*∼0.2-0.3 with *p*=0.001) indicating that the GI symptoms co-occur when present.

### 3.4. Crying Behavior

**Figure 1:**
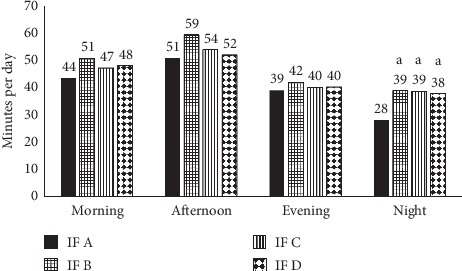
Crying/fussy behavior reported in subject diaries per IF. a denotes significant difference from IF *A*. IF *A* vs. IF *Bp*=0.004; IF A vs. IF *Cp*=0.026; IF *A* vs. IF *Dp*=0.002.

Figure 1 shows the mean duration of reported crying events at each period of the day per product group. The average crying time peaked in the afternoon and declined at night. Infants in the IF *A* group cried less at night time than infants in groups IF *B* (*p*=0.004), IF *C* (*p*=0.026), and IF *D* (*p*=0.002).

### 3.5. Heatiness Outcomes

The reports for heatiness for all product groups were similar across the 7 days. The groups were comparable for all symptoms of “heatiness” except dry faeces/yellow urine and sleeping problems (Table 6) with majority of the parents scoring their children in the “none/normal” category for the heatiness symptoms and thereby as having “no heatiness”. There were no reports of “moderate” or “severe” heatiness. IF *A*-fed infants showed lower odds compared to IF *B*, IF *C,* and IF *D* for the “heatiness” symptoms of dry faeces/yellow urine (all *p* < 0.0001) and sleeping problems (all *p* < 0.0001) (Table 7).

## 4. Discussion

This seven-day cross-sectional observational study showed that healthy, term infants fed IF *A* reported favourable stool characteristics and less night time crying than infants who consumed the other three commercially available formulas. Higher percentage of parents and/or caregivers of infants in the IF *A*-fed group reported absence or no complaints of abdominal distension, burping, flatulence, diarrhea, and constipation. Additionally, the study showed lower reports for dry faeces/yellow urine and sleeping problems, symptoms for “heatiness” as per TCM, in the IF *A* group as compared to the other groups.

In line with other reports [1, 3, 4], the majority of the infants in the present study were reported to suffer from one or multiple “minor” GI symptoms. While not harmful for the infant, these can be a matter of concern for the parents as the affected infants experience, at least, a moment of distress and thereby, impacting the quality of life for both infants and the parents [2, 6, 22]. There was no difference in frequency and quantity of formula feeding or defecation frequency amongst the infants on different formulas. The average daily defecation frequency (approx. 1.5 times per day) for all formula groups is comparable to that reported by den Hertog et al. for breastfed infants at 3 months from one of the largest cohorts of healthy infants [26]. The authors from this cohort also reported more yellow-coloured stools for breastfed infants and green-coloured stools as standard for formula-fed infants. In our study, over 80% of the IF *A*-fed infants had yellow/golden or orange-coloured stool comparable to that found in literature for breastfed infants [25, 26]. In contrast, stool colour for infants fed IF *B*, IF *C,* and IF *D* was largely orange (32–42%) or green (38–40%).

In our study, we also found a higher percentage of soft/formed stools in IF *A*-fed infants and a lower percentage of both watery and hard stools compared to IF *B*, IF *C*, and IF *D*. It is known that stool consistency can vary according to what an infant is fed (breast milk, type of formula [27, 28], type of solid food [29]), and stools tend to become harder as infants and children get older [29–31]. In exclusively breastfed infants at one month of age, stool is typically mushy, although watery stools can occur [32]. From birth to six months of age, runny and pasty stools occur with equal frequency, while liquid stools occur on occasion [26, 32]. Formula-fed infants typically report more soft and/or formed stools and less watery stools [26], as is also seen in this study.

Clear differences in the GI comfort symptoms between different formulas as well as a reduced crying time, particularly at night, were observed in the current study. Crying behavior in infants is considered to reflect basic, instinctive responses [33] that may be linked to separation, hunger, or other physical distress such as abdominal discomfort [34]. Not unexpectedly, the duration of crying in infants is inversely correlated with the duration of sleep time [34, 35]. Interestingly, less hard stools and less night time crying/fussiness are also reflected in the “Heatiness” symptoms of dry faeces/yellow urine and sleep problems. Although crying is a common spontaneous infant behavior, it can induce parental concern. Reduced crying time as seen with IF *A* in this study can, therefore, be an important and relevant benefit to improve quality of life.

One plausible explanation for the favourable effect of IF *A* on stool characteristics, GI symptoms, and crying time may be the minimal processing, as indicated by the low BL levels of this formula [11, 15, 16]. Maintaining Maillard reaction products as low as technically feasible is acknowledged as having a role in protein digestibility [15, 16]. A recent systematic review by van Lieshout GAA et al. concludes that glycation decreases protein digestibility and lowers amino acid availability, especially for lysine [36]. Undigested glycated protein can escape intestinal digestion and end up in the distal ileum and colon. This exposes the immature gut to intact proteins, which could potentially result in sensitization of the immune system, a possible allergic reaction as well as an unfavourable gut microflora composition and diarrhea [37, 38]. Additionally, reports by some authors indicate towards colonic fermentation of the undigested protein leading to the production of potentially unfavourable metabolites such as branched-chain fatty acids, ammonia, phenol, p-cresol, indole, and hydrogen sulfide [39–42] as well as some GI symptoms such as bloating and diarrhea [2]. In view of the infant's immature GI system, there is a higher relevance and need to ensure lower Maillard reaction products in the infant formula [12, 17–19]. According to van de Heijning et al. a formula specially designed with easily digestible proteins can be a good option for addressing mild GI symptoms in infants [2].

As the four infant formulas used in the study are from different manufacturers and commercially available, care was taken to ensure all selected formulas were vegetable fat-blend based, containing (prebiotic) fibres and with no probiotics-factors that are known to influence GI symptoms. We acknowledge that other than differences in BL levels, there are other compositional variations between the products such as different types of (prebiotic) carbohydrates. Similar favourable results on stool characteristics and GI symptoms including constipation are reported in the literature for IF with GOS, FOS and/or in combination with PDX, but it is not clear whether one is more effective than the other [43–46]. This suggests that the observed results may be associated with the potential differences in processing as indexed by BL levels or the different types of carbohydrates used in the formula.

To our knowledge, this is the first study to assess the occurrence of GI symptoms and crying behavior in Chinese infants in relation to different formula in a real-life setting. The strength of our study lies in consistent favourable reporting by parents over the seven-day period in a considerable sample size. This is seen by the good correlation between various GI symptoms indicating internal consistency and thereby, good quality of data. However, the current study design (cross-sectional observational study) also acts as a limitation since no causal relationship can be established. In addition, the stool consistency data (e.g., the incidence rate of hard and watery stools) relied on parental reports only and thus may be more prone to reporting biases compared with more objective methods. It is, however, unlikely that parents would underreport undesirable incidences. Detailed instructions (including photographic examples of stools corresponding to each point on the scale) were provided to parents to facilitate the completion of subject and stool diaries. We recognize that the inclusion of breastfed infants as a reference group as well as objective assessments may allow for direct comparisons with results from RCTs. Nevertheless, the current study clearly highlights the need to further explore the potential health benefits that can be accomplished with a minimally processed infant formula with low levels of Maillard reaction products [47].

## Figures and Tables

**Table 1:** Infant formula composition.

Formula	IF *A*	IF *B*	IF *C*	IF *D*
Energy (kJ)	2109	2146	2150	2100
Protein (g)	11.5	10.6	10.4	10.7
Fat (g)	26.4	28.1	28	27
Carbohydrates (g)	53.6	52.9	54	54
Galacto-oligosaccharides (GOS)	3	3.2	—	1.53
Fructo-oligosaccharides (FOS)	—	—	2.3	—
Polydextrose (PDX)	—	—	—	1.56
Iron (mg)	6	5.4	6.2	5.1
Blocked lysine (BL) (%)	9	12	11	20

All values except BL are per 100 g powder as declared on label; BL are analyzed values.

**Table 2:** Baseline characteristics per group.

Characteristics	IF *A* (*n* = 105)	IF *B* (*n* = 100)	IF *C* (*n* = 103)	IF *D* (*n* = 101)
Infants				
Gestational age (wks)	39.2 ± 1.3	39.1 ± 1.3	39.2 ± 1.3	39.3 ± 1.3
Birth weight (kg)	3.39 ± 0.36	3.39 ± 0.38	3.30 ± 0.38	3.31 ± 0.38
Apgar score	10 (6–10)	10 (8–10)	10 (8–10)	10 (9–10)
Age at enrolment (days)	93.5 ± 24.6	99.2 ± 21.4	98.1 ± 22.4	95.0 ± 26.9
Weight at enrolment (kg)	6.08 ± 0.91	6.28 ± 0.83	6.28 ± 0.87	6.17 ± 0.97
Height at enrolment (cm)	60.4 ± 3.5	61.1 ± 3.7	60.7 ± 3.4	60.5 ± 3.5
Delivery (% vaginal)	61	59	59.2	58.4
Gender (% male)	56	45	48	59

Maternal				
Education (% bachelor's degree)	46.7	33	47.6	34.7
Employed (%)	88.6	87	89.3	84.2

Paternal				
Education (% bachelor's degree)	46.7	40	49.5	45.5
Employed (%)	98.1	100	100	99

Socioeconomics				
Living area (%)				
60–90 m^2^	40	33.0	39.8	46.5
90–120 m^2^	34.3	47.0	47.6	37.6

Family income per month				
6000–8000 RMB	26.7	24	16.5	22.8
More than 8000 RMB	70.5	73	80.6	71.3

**Table 3:** Feeding habits per group.

Variables	IF *A* (*n* = 105)	IF *B* (*n* = 100)	IF *C* (*n* = 103)	IF *D* (*n* = 101)
Formula feeding start age (day)	34.7 ± 29.7	36.0 ± 33.3	43.5 ± 27.4	43.3 ± 32.3
Daily feeding frequency (times)	5.7 ± 0.9	5.9 ± 0.8	5.8 ± 0.8	5.9 ± 0.7
Feeding volume (ml/day)	723 ± 93	725 ± 96	726 ± 88	726 ± 93
Main caregiver (% mother)	99	97	100	97

**Table 4:** Stool characteristics and gastrointestinal symptoms per group.

Variables	IF *A* (*n* = 105)	IF *B* (*n* = 100)	IF *C* (*n* = 103)	IF *D* (*n* = 101)	*p* value
*Amsterdam stool scale (7-day average)*					
Daily stool frequency (mean score)	1.50 ± 0.75	1.39 ± 0.64	1.52 ± 0.85	1.60 ± 0.99	0.312
Daily stool amount (mean score)	2.44 ± 0.74	2.32 ± 0.72	2.46 ± 0.79	2.45 ± 0.78	0.117
Stool amount (# reports)					
0-No stool	4	5	5	5	
1-smear	8	9	11	8	
2-up to 25%	49	51	39	45	
3-25–50%	37	30	41	34	
4->50%	7	5	7	9	
Daily stool consistency (mean score)	1.44 ± 0.67	1.16 ± 0.78	1.19 ± 0.79	1.22 ± 0.81	0.0001^*∗∗∗*^
Stool consistency (# reports)					
0-No stool	4	5	5	5	
1-watery	7	14	14	12	
2-soft/Formed	91	72	75	73	
3-hard	3	9	10	11	
Daily stool colour (mean score)	2.09 ± 0.67	2.74 ± 1.08	2.53 ± 0.79	2.32 ± 0.75	0.0008^*∗∗∗*^
Stool colour (# reports)					
No stool	4	5	5	5	
I (yellow/golden)	13	8	6	13	
II (orange)	69	32	43	41	
III (green)	16	40	40	39	
IV (brown)	2	7	8	2	
V (grey/black)	1	5	1	0	
VI (white)	0	3	0	0	

*Gastrointestinal symptoms based on the subject diary (7-day average)*					
Abdominal distension (mean score)	1.22 ± 0.82	1.48 ± 0.82	1.43 ± 0.75	1.44 ± 0.72	0.001^*∗*^
Burping (mean score)	1.39 ± 0.96	1.66 ± 0.90	1.85 ± 0.93	1.78 ± 0.91	0.004^*∗*^
Flatulence (mean score)	1.07 ± 0.83	1.45 ± 0.72	1.36 ± 0.67	1.55 ± 0.76	<0.0001^*∗∗∗*^
Diarrhea (mean score)	0.88 ± 0.67	1.18 ± 0.63	1.15 ± 0.56	1.08 ± 0.50	<.0001^*∗∗∗*^
Constipation (mean score)	0.92 ± 0.75	1.11 ± 0.51	1.38 ± 0.92	1.20 ± 0.66	<.0001^*∗∗∗*^
Colic (mean score)	1.13 ± 0.45	1.13 ± 0.48	1.05 ± 0.28	1.08 ± 0.37	0.167
Regurgitation (mean score)	0.14 ± 0.43	0.08 ± 0.29	0.08 ± 0.29	0.09 ± 0.32	0.648
Vomiting (mean score)	0.28 ± 0.63	0.29 ± 0.71	0.26 ± 0.59	0.22 ± 0.52	0.935

*# Reports “no complaints”*					
Abdominal distension	16.2	0	0	2.4	
Burping	17.9	0	0	1.6	
Flatulence	24	0.4	0	2.7	
Diarrhea	27	5.2	4.1	5.8	
Constipation	26.7	5	3.6	5.6	
Colic	0.1	0.3	0	0.7	
Regurgitation	83.4	80.1	82.7	82.6	
Vomiting	93.4	92.7	95.4	91.8	

^*∗*^ *p* < 0.05; ^*∗∗*^*p* < 0.001; ^*∗∗∗*^*p* < 0.0001. Mean score for GI symptoms based on 0: no complaints, 1: very mild, 2: mild, 3: moderate, 4: quite severe, 5: very severe.

**Table 5:** Pairwise comparison of product effect on stool characteristics, gastrointestinal, and heatiness symptoms.

	IF *A* vs IF *B*		IF *A* vs IF *C*		IF *A* vs IF *D*		IF *B* vs IF *C*		IF *B* vs IF *D*		IF *C* vs IF *D*	
	OR (95% CI)	*p* value	OR (95% CI)	*p* value	OR (95% CI)	*p* value	OR (95% CI)	*p* value	OR (95% CI)	*p* value	OR (95% CI)	*p* value
Stool characteristics												
Stool consistency	2.58 (1.78, 3.75)	0.0001^*∗∗∗*^	2.39 (1.69, 3.39)	<0.0001^*∗∗∗*^	2.07 (1.36, 3.16)	0.0007^*∗∗∗*^	0.93 (0.62, 1.83)	0.707	0.80 (0.50, 1.29)	0.359	0.87 (0.54, 1.38)	0.544
Stool colour	0.25 (0.15, 0.40)	<0.0001^*∗∗∗*^	0.50 (0.34, 0.75)	0.0006^*∗∗∗*^	0.57 (0.37, 0.88)	0.008^*∗∗*^	2.01 (1.30, 3.12)	0.002^*∗*^	2.27 (1.43, 3.60)	0.0005^*∗∗∗*^	1.13 (0.76, 1.68)	0.552

Gastrointestinal symptoms												
Abdominal distension	0.51 (0.34, 0.77)	0.002^*∗*^	0.55 (0.38, 0.81)	0.002^*∗*^	0.51 (0.34, 0.77)	0.001^*∗∗*^	1.09 (0.71, 1.66)	0.699	1.01 (0.64, 1.58)	0.977	0.93 (0.61, 1.40)	0.717
Burping	0.60 (0.45, 0.80)	0.004^*∗∗*^	0.38 (0.27, 0.54)	<0.0001^*∗∗∗*^	0.44 (0.31, 0.62)	<0.0001^*∗∗∗*^	0.64 (0.44, 0.91)	0.015^*∗*^	0.73 (0.50, 1.05)	0.089	1.14 (0.75, 1.73)	0.529
Flatulence	0.28 (0.17, 0.44)	<0.0001^*∗∗∗*^	0.36 (0.25, 0.54)	<0.0001^*∗∗∗*^	0.21 (0.14, 0.32)	<0.0001^*∗∗∗*^	1.29 (0.87, 1.92)	0.206	0.74 (0.50, 1.10)	0.14	0.57 (0.40, 0.81)	0.002^*∗*^
Diarrhea	0.25 (0.17, 0.36)	<0.0001^*∗∗∗*^	0.26 (0.17, 0.38)	<0.0001^*∗∗∗*^	0.33 (0.23, 0.48)	<0.0001^*∗∗∗*^	1.04 (0.73, 1.49)	0.83	1.36 (0.99, 1.87)	0.062	1.31 (0.94, 1.81)	0.107
Constipation	0.36 (0.25, 0.52)	<0.0001^*∗∗∗*^	0.20 (0.13, 0.31)	<0.0001^*∗∗∗*^	0.29 (0.19, 0.43)	<0.0001^*∗∗∗*^	0.56 (0.42, 0.75)	0.0001^*∗∗∗*^	0.80 (0.60, 1.05)	0.106	1.41 (1.03, 1.94)	0.033^*∗*^

^*∗*^ *p* < 0.05; ^*∗∗*^*p* < 0.001, ^*∗∗∗*^*p* ≤ 0.0001. Results presented for outcomes with significant product effects in type 3 GEE analysis. Multiple comparisons with Bonferroni adjustment.

**Table 6:** Self-reported heatiness symptoms per group.

Symptoms	IF *A* (*n* = 105)	IF *B* (*n* = 100)	IF *C* (*n* = 103)	IF *D* (*n* = 101)	*p* value
Dry faeces, yellow urine (% normal stool frequency)	82.9	64.0	68.0	66.3	<0.0001^*∗∗∗*^
Aphtha and dry mouth (% none)	98.1	99.0	98.1	97.0	0.124
Sleeping problem (% sleep normally)	85.7	54.0	49.5	57.4	<0.0001^*∗∗∗*^
Eye boogers (% normal)	89.5	90.0	84.5	91.1	0.295
Dry skin (% normal)	91.4	90.0	88.4	91.1	0.476
Bleeding nose (% none)	97.1	96.0	99.0	99.0	n.a.
Dry cough (% none)	93.3	89.0	90.3	92.1	0.866
Throat pain (% none)	97.1	95.0	92.2	95.1	0.903
Palm temperature (% cool and wet)	82.9	84.0	86.4	86.1	0.256
Anus colour (% pink)	88.6	84.0	86.4	91.1	0.640
Bad breath (% none)	98.1	96.0	97.1	97.0	0.482

Percentage reported is for Day 1.

**Table 7:** Pairwise comparison of product effect on heatiness symptoms.

	Dry faeces, yellow urine		Sleeping problem	
	OR (95% CI)	*p* value	OR (95% CI)	*p* value
IF *A* vs IF *B*	0.51 (0.38, 0.68)	<0.0001^*∗∗∗*^	0.21 (0.14, 0.33)	<0.0001^*∗∗∗*^
IF *A* vs IF *C*	0.41 (0.29, 0.59)	<0.0001^*∗∗∗*^	0.19 (0.13, 0.28)	<0.0001^*∗∗∗*^
IF *A* vs IF *D*	0.39 (0.29, 0.55)	<0.0001^*∗∗∗*^	0.23 (0.15, 0.34)	<0.0001^*∗∗∗*^
IF *B* vs IF *C*	0.81 (0.59, 1.09)	0.166	0.90 (0.66, 1.81)	0.529
IF *B* vs IF *D*	0.78 (0.60, 1.01)	0.058	1.06 (0.75, 1.49)	0.746
IF *C* vs IF *D*	0.96 (0.68, 1.36)	0.827	1.17 (0.87, 1.57)	0.29

^*∗*^ *p* < 0.05, ^*∗∗*^*p* < 0.001, and ^*∗∗∗*^*p* ≤ 0.0001. Results presented for outcomes with significant product effect in Type 3 GEE Analysis. Multiple comparisons with Bonferroni adjustment.

## Data Availability

The data used to support the findings of this study are available from the corresponding author upon request.
